# 

**Published:** 1950-04

**Authors:** 


PUBLISHED UNDER THE AUSPICES OF THE BRISTOL MEDICO-CHIRURGICAL SOCIETY

### THE BRISTOL MEDICO-CHIRURGICAL JOURNAL

*A Journal of the Medical Sciences for the*

### WEST OF ENGLAND AND SOUTH WALES

ARMY MEDICAL OCT 27 1950 LIBRARY

*Editor:*

E. WATSON-WILLIAMS, *M.C.*, M.D., Ch.M., F.R.C.S.

*with whom are associated*

G. E. F. SUTTON, *M.C.*, M.D., F.R.C.P.

J. H. MIDDLEMISS, M.D., D.M.R.D., F.F.R.

N. S. CRAIG, *M.C.*, M.D., *Assistant Editor.*

*Local Correspondents:*

BATH—A. D. BATEMAN, *O.B.E.*, F.R.C.S.

BOURNEMOUTH—A. MACKEZIE ROSS, M.D.

CARDIFF—J. D. SPILLANE, M.D., M.R.C.P. EXETER—L. N. JACKSON, *M.C.*, D.M.

GLOUCESTER—R. F. JARRETT, M.B., M.R.C.P.

NEWPORT—J. L. D. WILLIAMS, M.B., F.R.C.S.

PLYMOUTH—C. R. CROFT, D.M., M.R.C.P. TAUNTON—Dr. M. MCALLEY, D.M.R.

TORQUAY—Dr. A. ROBINSON THOMAS, D.M.R.E.

TRURO—Dr. F. D. M. HOCKING, F.R.I.C. YEOVIL—J. A. VERE NICOLL, F.R.C.S.

BRISTOL:
J. W. ARROWSMITH LTD., QUAY STREET AND SMALL STREET

Vol. LXVII. No. 242. *Price:* FOUR SHILLINGS net. APRIL · 1950

